# The Urgent Need for Cardiometabolic Health Training: A Call to Action

**DOI:** 10.7759/cureus.103268

**Published:** 2026-02-09

**Authors:** Khalid Sawalha

**Affiliations:** 1 Cardiovascular Disease, University of Arkansas for Medical Sciences, Little Rock, USA; 2 Cardiometabolic Medicine, University of Missouri Kansas City School of Medicine, Kansas City, USA

**Keywords:** cardiology, cardiometabolic health, diabetes, endocrine, fellowship training, hypertension

## Abstract

Since Reaven’s 1988 observation linking insulin resistance to both type 2 diabetes mellitus (T2DM) and cardiovascular diseases (CVD), additional abnormalities have been identified, later described collectively as metabolic syndrome (MetS), distinct from the previously termed cardiac syndrome X. Although inflammation is now recognized as central to these metabolic disturbances, components such as obesity, insulin resistance, dyslipidemia, and hypertension have long been associated with what we now call MetS.

Despite evolving definitions of MetS, its clinical utility lies in identifying patients at elevated risk of CVD. Unfortunately, the incidence of MetS parallels the rising prevalence of both obesity and T2DM. While dietary and lifestyle interventions show promise at the individual level, more is needed from a healthcare systems perspective. The concept of cardiometabolic health (CMH) has recently emerged to raise awareness and guide best practices in promoting cardiovascular health and preventing adverse outcomes. Additionally, CMH aims to identify disparities in both diagnosis and care delivery, regardless of age, sex, ethnicity, or socioeconomic status.

This effort is timely, as the decline in CVD mortality has stagnated, raising concerns about worsening risk factors or inequitable care access. Although not all pathophysiologic mechanisms linking MetS, T2DM, and CVD are fully elucidated, substantial evidence implicates genetic, epigenetic, environmental, and lifestyle factors. Particularly concerning are rising MetS rates among young adults and increasing prevalence in Hispanic and Asian populations. A central obstacle to addressing MetS, T2DM, and CVD is obesity.

Given the demands of endocrinology and cardiology training programs, a new interdisciplinary field, cardiometabolic medicine (CMM), has emerged to address the complex needs of patients with overlapping cardiometabolic disorders. Although still evolving, CMM is essential for training future clinicians to address these escalating health challenges. Metabolic syndrome is a diagnostic risk construct, whereas cardiometabolic medicine is a clinical and training framework that incorporates MetS when present but extends beyond it to deliver integrated, life-course cardiovascular-renal-metabolic prevention and treatment.

## Editorial

The need for cardiometabolic medicine training

The American College of Cardiology Core Cardiovascular Training Statement (COCATS 4), Task Force 2, specifies that only one month of training in preventive cardiovascular medicine - either as a dedicated rotation or dispersed over three months - is required to meet Level I training standards. Moreover, this document provides no specific requirements for achieving Level II or III proficiency. Similarly, the Accreditation Council for Graduate Medical Education (ACGME) program requirements for Endocrinology, Diabetes, and Metabolism fellows state that trainees “must demonstrate competence in the practice of health promotion, disease prevention, diagnosis, care, and treatment of patients of each gender, from adolescence to old age, during health and all stages of illness” [1]. The requirements also reference the “prevention and surveillance of microvascular and macrovascular complications” [1].

Clearly, these limited requirements do not encompass the breadth of knowledge necessary to effectively diagnose and manage cardiometabolic health (CMH). Current curricula remain largely focused on the estimation of atherosclerotic cardiovascular disease (ASCVD) risk to predict myocardial infarction or stroke. The primary training gap is not recognition of metabolic syndrome (MetS) but the lack of structured education to manage cardiometabolic disease as an integrated, multisystem continuum. Despite MetS affecting approximately 33%-35% of U.S. adults and fewer than 7% meeting criteria for optimal cardiometabolic health, cardiology and endocrinology training remains largely siloed and prevention-limited.

Such assessments are typically performed during routine clinical encounters using readily available risk calculators. However, while these tools estimate future myocardial infarction risk, they fail to capture the broader complexity of cardiometabolic disease, which is also associated with atrial fibrillation, heart failure, obstructive sleep apnea, chronic kidney disease, and chronic liver disease [2,3].

Therefore, this document aims to highlight the growing path of cardiometabolic training with a goal of exposing trainees to a comprehensive curriculum, even in the absence of established standards, to develop essential skills for addressing cardiometabolic disease (CMD) and achieving optimal CMH. This training will help clinicians better understand the interconnections between disease processes and enable earlier identification, diagnosis, and treatment, often before patients are referred to endocrinologists or cardiologists.

In an attempt to address the urgent cardiometabolic health needs, a unique cardiometabolic fellowship training program was established and launched at the University of Missouri-Kansas City in 2022, following collaborative efforts among multiple divisions within the Department of Internal Medicine, including Cardiovascular Medicine, Endocrinology and Diabetes, Obesity Medicine, Nephrology, Sleep Medicine, and PharmD. This program can serve as a strong model for other institutions aiming to address urgent CMH needs [4]. Early program experience demonstrates feasibility and strong trainee engagement, with fellows achieving multidisciplinary clinical exposure, increased use of evidence-based cardiometabolic therapies, and development of cross-specialty care pathways, supporting the program’s scalability as a training model. 

To provide practical context for this call to action, this Editorial outlines briefly the structure of the Cardiometabolic Medicine fellowship established at the University of Missouri-Kansas City. This is a one-year, non-ACGME program designed for physicians who have completed training in internal medicine or family medicine. The fellowship provides structured clinical rotations across cardiology, endocrinology, sleep medicine, weight management, and nephrology clinics. The fellowship’s learning objectives include mastery of comprehensive cardiometabolic risk assessment, competence in managing diabetes, interpret continuous glucose monitoring devices (CGM) data, dyslipidemia, hypertension, and obesity within a cardiovascular prevention framework, proficiency in prescribing and monitoring advanced cardiometabolic therapies (e.g., sodium-glucose cotransporter 2 (SGLT2) inhibitors, glucagon-like peptide-1 (GLP-1) receptor agonists), the use of insulin pumps, and engagement in clinical research and community outreach.

Rotations include exposure to different specialties and can be customized based on the fellow’s interests. They consist of three months of inpatient cardiology and endocrinology consults; two months of cardiology and endocrinology procedures, such as surface and transesophageal echocardiograms, coronary calcium scoring, and insulin pump management; two months in sleep medicine clinics; two months in weight management clinics; two months in nephrology clinics; and one month of elective rotation. This program enables trainees to integrate risk factor assessment and management in a manner not typically offered in traditional endocrinology or cardiology fellowships.

In addition to the core curriculum, fellows have the flexibility to pursue their clinical interests, including advanced imaging and diagnostics. For example, based on the interest of the first fellow in cardiovascular disease, he was tasked with reading 150 electrocardiograms (ECGs) and 150 coronary artery calcium scoring CTs. He also performed and interpreted 50 transthoracic and 10 transesophageal echocardiograms under supervision. Fellows actively see patients in outpatient clinics and inpatient consult services, where they conduct independent evaluations and present to attending physicians for supervision and collaborative care planning.

The fellowship also includes a research training component, offering fellows with opportunities to engage in population health initiatives and explore novel treatments, risk models, and innovative approaches to CMH. Additionally, fellows complete a graduate-level course in Biostatistics 1 (MEDB 5501), which may be applied towards a certificate or master’s degree in clinical research, further enhancing their academic development.

As cardiometabolic medicine continues to evolve and gaps in the current system of care become more evident, the urgency for a dedicated cardiometabolic specialty that provides both the knowledge and skills to manage these complex conditions grows.

Why is cardiometabolic medicine training necessary? First, the global surge in obesity and type 2 diabetes mellitus (T2DM) demands dedicated expertise [5]. There simply aren’t enough endocrinologists to manage this growing population. A specialist focused on CMH would also be better equipped to overcome barriers to addressing obesity in the general population. In the United States, cardiometabolic health has worsened over recent decades, without fully meeting the criteria for optimal cardiometabolic health and substantial disparities by age, sex, education, and ethnicity [5]. Globally, the rapid rise in obesity and type 2 diabetes - affecting hundreds of millions of adults and children - highlights the broader workforce implications and reinforces the need for scalable cardiometabolic training models [5].

Second, data from the United States indicate worsening CMH between 1999-2000 and 2017-2018, accompanied by persistent disparities in age, sex, education, and ethnicity [5]. A generation born in the late 1970s and early 1980s, continuously exposed to obesogenic environments, is now experiencing significant metabolic complications, particularly among socially and economically vulnerable groups [5]. Furthermore, in 2012, the United States reported a 35% increase in MetS prevalence compared to the 1980s. Since approximately 85% of patients with T2DM also have MetS, this population faces a markedly elevated cardiovascular risk. The scope is alarming: in 2017, 12.2% of U.S. adults had T2DM, and given that MetS prevalence is roughly threefold higher, an estimated one-third of the adult population is affected. In addition, as of 2015, 604 million adults and 108 million children globally were obese, based on data spanning 195 countries. Since 1980, obesity rates have doubled in many nations, with pediatric cases rising sharply. Thus, achieving optimal CMH must be a critical public health priority. 

Third, the emergence of SGLT2 inhibitors and GLP-1 receptor agonists, agents shown to improve cardiovascular and renal outcomes, was initially met with underuse due to provider unfamiliarity with patient selection and follow-up. Providers trained in CMD would be more confident in initiating these therapies and managing follow-up appropriately.

A board of cardiometabolic medicine with formal certification is now warranted to standardize training, credential physician expertise, and close the documented gap between siloed cardiology/endocrinology education and training.

Finally, as Desiderius Erasmus said, “prevention is better than cure.” By the time patients reach endocrinologists or cardiologists, opportunities for effective primary prevention may be missed. Non-specialists unfamiliar with cardio-renal-metabolic strategies are less likely to implement them.

## References

[1] (2025). ACGME Program Requirements for Graduate Medical Education in Endocrinology, Diabetes, and Metabolism. https://www.acgme.org/globalassets/pfassets/programrequirements/2025-reformatted-requirements/143_endocrinologydiabetesmetabolism_2025_reformatted.pdf.

[2] Sawalha K, Norgard N, López-Candales A (2023). Epigenetic regulation and its effects on aging and cardiovascular disease. Cureus.

[3] Sawalha K, Tripathi V, Alkhatib D, Alalawi L, Mahmood A, Alexander T (2023). Our hidden enemy: ultra-processed foods, inflammation, and the battle for heart health. Cureus.

[4] Sawalha K, Asad R, Tadisina S, López-Candales A, Asif T (2026). Addressing the urgent cardiometabolic health needs through a unique cardiometabolic fellowship training program. Circulation: Population Health and Outcomes.

[5] Almohtasib Y, Fancher AJ, Sawalha K (2024). Emerging trends in atherosclerosis: time to address atherosclerosis from a younger age. Cureus.

